# Recurring battle with Dengue; post heavy monsoon in Pakistan

**DOI:** 10.1016/j.amsu.2022.104400

**Published:** 2022-08-17

**Authors:** Muttia Abdul Sattar, Hadia Nadeem

**Affiliations:** Karachi Medical and Dental College, Pakistan

Dengue is a mosquito-borne viral infection transmitted to humans, caused by four types of dengue virus (DENV) (DENV-1, DENV-2, DENV-3, DENV-4) and primarily carried by the species *Aedes aegypti* [1]. It presents with a high fever accompanied by other flu-like mild symptoms that include severe headache, pain behind the eyes, muscle and joint pains and vomiting. However, the diseased person may enter a potentially fatal complication, severe dengue also known as dengue haemorrhagic fever (DHF), which is a leading cause of serious illness and death in some Asian and Latin American countries [1]. DHF can cause serious bleeding, a sudden drop in blood pressure and death [2], and the warning signs for this critical phase include severe abdominal pain, persistent vomiting and rapid breathing [1].

Pakistan first reported an epidemic of Dengue fever in 1994 and since November 2005 annual recurrence has become apparent [3]. According to WHO dengue is now endemic in Pakistan, with the last notable outbreak, with 53,498 cases and 95 deaths being reported between September to December 2019 [4]. It circulates throughout the year, peaking in the post monsoon period [3], as the stagnant rainwater provides a good enough breeding ground for female mosquitoes to lay their eggs. Over the current 2022 monsoon season, Pakistan has experienced a heavy amount of rainfall. According to Pakistan Meteorological Department, it was the wettest July since 1961 with rainfall 180% above average [5], resulting in flash and urban flooding throughout Pakistan. Moreover, a tendency of above normal precipitation is predicted for most parts of the country during August 2022, which can trigger further flooding [6].

This higher than usual monsoon rainfall raises an alarming concern for a potential outbreak of dengue fever in the country. Pakistan is already dealing with 6th wave of COVID-19 and an outbreak of Cholera [7], hence another massive outbreak will be a serious threat to the fragile health care system of the country. Therefore, we aim to highlight the safety measures that should be taken by the concerned authority immediately. For instance, to prevent mosquitoes breeding, water pumps and mobile hydrants should regularly collect stagnant rainwater from areas in the cities where there is a poor drainage system. There should be insecticide spraying by the government in the affected cities. Awareness on a potential spike in dengue infection should be spread to the masses to take prevention on a personal level which includes applying mosquito repellents, using coils, bed nets and wearing full clothing with minimum skin exposure to protect oneself from mosquitoes. Hospitals should carry out active screening of suspected patients for early detection of the disease, reducing the risk of complications and restricting the advancement of the number of cases.

## Ethical approval

None required.

## Sources of funding

None.

## Author contribution

Muttia Abdul Sattar, Hadia Nadeem. All authors contributed the same.

## Conflicts of interest

None.

## Registration of research studies

Name of the registry:

Unique Identifying number or registration ID:

Hyperlink to your specific registration (must be publicly accessible and will be checked):

## Guarantor

Muttia Abdul Sattar.

## Consent

None required.
